# Exploring the mediating factors in the telework-mental health relationship: a cross-sectional analysis of the BELHEALTH study

**DOI:** 10.1136/bmjph-2025-003249

**Published:** 2026-02-18

**Authors:** Eduardo Antonio Bracho Montes de Oca, Robby De Pauw, Beatrijs Moerkerke, Lize Hermans, Camille Duveau, Kayleigh De Meulemeester, Barbara Cagnie, Bas de Geus

**Affiliations:** 1 IACCHOS, Université catholique de Louvain, Louvain-la-Neuve, Belgium; 2 Department of Rehabilitation Sciences, Ghent University, Gent, Belgium; 3 Department of Epidemiology and Public Health, Sciensano, Brussels, Belgium; 4 Department of Data Analysis, Ghent University, Gent, Belgium; 5 Institute of Health and Society, Université catholique de Louvain, Brussels, Belgium

**Keywords:** Mental Health, Occupational Medicine, Statistics as Topic

## Abstract

**Introduction:**

This study investigates the relationship between telework and mental health, focusing on mediating factors.

**Methods:**

A sample was drawn from the June 2023 wave of the BELHEALTH study, which monitors mental health trends in Belgium. The sample included 2323 employed participants aged 18–64 years. Interventional effects mediation analyses were conducted to explore the relationship between the frequency of telework (monthly, weekly, and daily) and mental health outcomes including anxiety (Generalised Anxiety Disorder-7, binary), depression (Patient Health Questionnaire-9, binary), burnout (Burnout Assessment Tool-12, scale 1–5) and work engagement (Utrecht Work Engagement Scale-3, scale 1–5) through the following mediators: workload, emotional load, role conflict, autonomy, social support and skills use.

**Results:**

Telework had both direct and indirect effects on mental health. The total effect of weekly telework on work engagement was —0.1614 (95% CI −0.2286 to –0.0972; p<0.01), indicating an overall decrease in work engagement when considering indirect and direct effects. While weekly telework was not significantly associated with anxiety and depression, it was directly associated with an average increase in burnout (0.1339, 95% CI 0.0875 to 0.1801; p<0.01), and a direct decrease in average work engagement (−0.2158, 95% CI −0.2783 to −0.1505; p<0.01). Indirectly, weekly telework was linked with burnout through various job demands and resources. For example, emotional load (−0.0427, 95% CI −0.0600 to –0.0272, p<0.01), and role conflict (−0.0266, 95% CI −0.0419 to −0.0122, p<0.05) were significant mediators of burnout.

**Conclusions:**

It is essential to consider the job characteristics of employees who telework, and the resources they have available to foster healthy workplaces.

**WHAT IS ALREADY KNOWN ON THIS TOPIC**

- Job characteristics, such as social support or workload, are determinants of work-related health indicators, such as work engagement and burnout, and it is hypothesised that telework modifies the job characteristics due to the change in work location.

**WHAT THIS STUDY ADDS**

- Telework appears to negatively impact work engagement when employees telework weekly.
- Telework does not affect anxiety nor depression.
- Taking all the mediators together, anxiety and depression seemed to be reduced in the weekly teleworker group compared with the non-teleworker group.

**HOW THIS STUDY MIGHT AFFECT RESEARCH, PRACTICE OR POLICY**

- To sustain work engagement during telework, employers should redesign jobs to strengthen positive job characteristics, by facilitating intermediate factors such as social support and autonomy.

## Introduction

 The traditional workplace has undergone a radical transformation in recent years due to the rise of telework, shifting from the traditional office to various spaces. According to Eurofound, 41.7 million people in Europe teleworked (here: worked from home) in 2021, doubling the number of teleworkers compared with 2020 which has been attributed to COVID-19. The prevalence of telework in 2021 varied across Europe, from 6% in Bulgaria to around 50% in the Netherlands, with a European prevalence of 22%. In Belgium, the prevalence of telework was 33%–40%.^1^

The rise of telework has raised questions about its impact on mental health. Data from the European Working Conditions Telephone Survey 2020–2021 suggests that people doing telework have a higher anxiety rate than the national average across the European Union, with the exception of Sweden and Czechia.^1^ Given that people usually spend more than half of their awake time at the workplace, it is important to understand the factors that determine whether telework (and factors within that job) is beneficial or harmful to mental health.

Unlike the earlier occupational stress theories such as the Demand-Control Model^2^ and the Effort-Reward Imbalance model^3^; Bakker, Demerouti and Schaufeli centralise two main job characteristics: job demands and job resources.^4^ Job demands are associated with physical, psychological and social aspects of costs (eg, workload, emotional demands). In contrast, job resources (eg, social support) refer to aspects of a job that help reduce physiological, psychological and social costs, or promote personal development. Moreover, job demands and job resources are related to two processes: the health-impairment and the motivational process, each predicting burn-out and work engagement, respectively.^4^ High job demands and lack of or insufficient job resources are related to burnout.^5^ These processes can predict long-term mental health outcomes.^6^

In a meta-analysis by Mazzetti *et al*, job resources and their impact on work engagement were investigated. They found that social support and autonomy (job control) were correlated with work engagement.^7^ Moreover, job demands and job resources are not only predictors of burnout and work engagement, respectively, but are also linked to other mental health outcomes. For instance, Bonde showed that a higher job demand is associated with a higher risk of depressive symptoms.^8^

Given that telework modifies the work location and temporal characteristics of work, it can be hypothesised that it changes the job demands and job resources, which in turn can directly or indirectly affect mental health (eg, burnout, work engagement). Job demands such as time pressure and role conflict seem to be reduced by telework, while job resources like autonomy are increased^9^ and social support is reduced.^9 10^ Similarly, Gajendran and Harrison found that perceived autonomy mediated the relationship of telework and role stress.^11^ These findings highlight the importance of the work context surrounding telework as a critical factor in understanding its impact on mental health.

Drawing from the affective event theory, the work environment (job characteristics or location) has been hypothesised to influence affective experiences at work.^12^ Given that telework increases the physical distance between employees themselves and their employers, it would be expected that there are fewer day-to-day interpersonal interactions with clients, colleagues and supervisors. As a result, fewer conflicting relationships would be expected, but social support may also be reduced. Moreover, employees engaged in highly emotionally demanding tasks may perceive telework as beneficial, as working from home offers greater control over the timing and nature of these interactions. Also, workload might be increased during telework as it can be seen as taking work to home.^13^ Lastly, working from home seems to be task-specific, and therefore there would be a need for different skills. To identify the conditions under which telework may be beneficial for employees, it is therefore important to explore the underlying mechanisms linking telework and mental health.

Most studies examining mediation and effect modification in the telework and mental-health relationship assume the absence of paths between job demands and job resources. However, it can be argued that job demands and job resources influence one another (for instance, in the model (eg, workload affects emotional load), as stated by Bakker and Demerouti).^9^,^1114^ In this paper, we explore the telework-mental health relationship without making assumptions about the underlying causal relationship between the mediators. We hypothesise that telework impacts mental health outcomes (anxiety, depression, burnout and work engagement) both directly and indirectly through mediators such as workload, emotional load, role conflict, autonomy, social support and skills use.

## Methodology

### Study design

This study is a cross-sectional analysis embedded within the Belgian Health and Well-being study (BELHEALTH), an ongoing cohort initiated in 2022 to monitor the mental health trends over time in Belgium.^17 18^ Cohort members receive an email invitation to complete an online questionnaire every 4 months, which captures demographic characteristics, work situation and mental health. In the third wave (21 June 2023 to 6 July 2023), thematic questions related to work context (occupation, sector, type of contract) were included. The BELHEALTH participants were initially recruited among the Belgian COVID-19 health surveys; details of the cohort design and methodology have been reported elsewhere, see Braekman *et al* and the project website.^18^,^20^ All persons of 18 years and older residing in Belgium could fill in the COVID-19 health surveys. A total of 11 560 individuals agreed to join the BELHEALTH cohort. In the second wave of BELHEALTH (February 2023), additional participants were recruited through the Belgian National Register to obtain a more representative sample of the Belgian population (18+). Postal letters were sent to 21 046 persons targeting in particular young people, men and people living in Wallonia. A total of 1162 individuals from this sample participated in wave 2 (response rate=5,5%), and 785 of them provided a valid email address and agreed to become a cohort member. In wave 3 (June 2023), a total of 7026 individuals participated in the survey.

The reporting of this study followed the Strengthening the Reporting of Observational Studies in Epidemiology reporting guidelines (see online supplemental material 1).^21^

**Figure 1:**
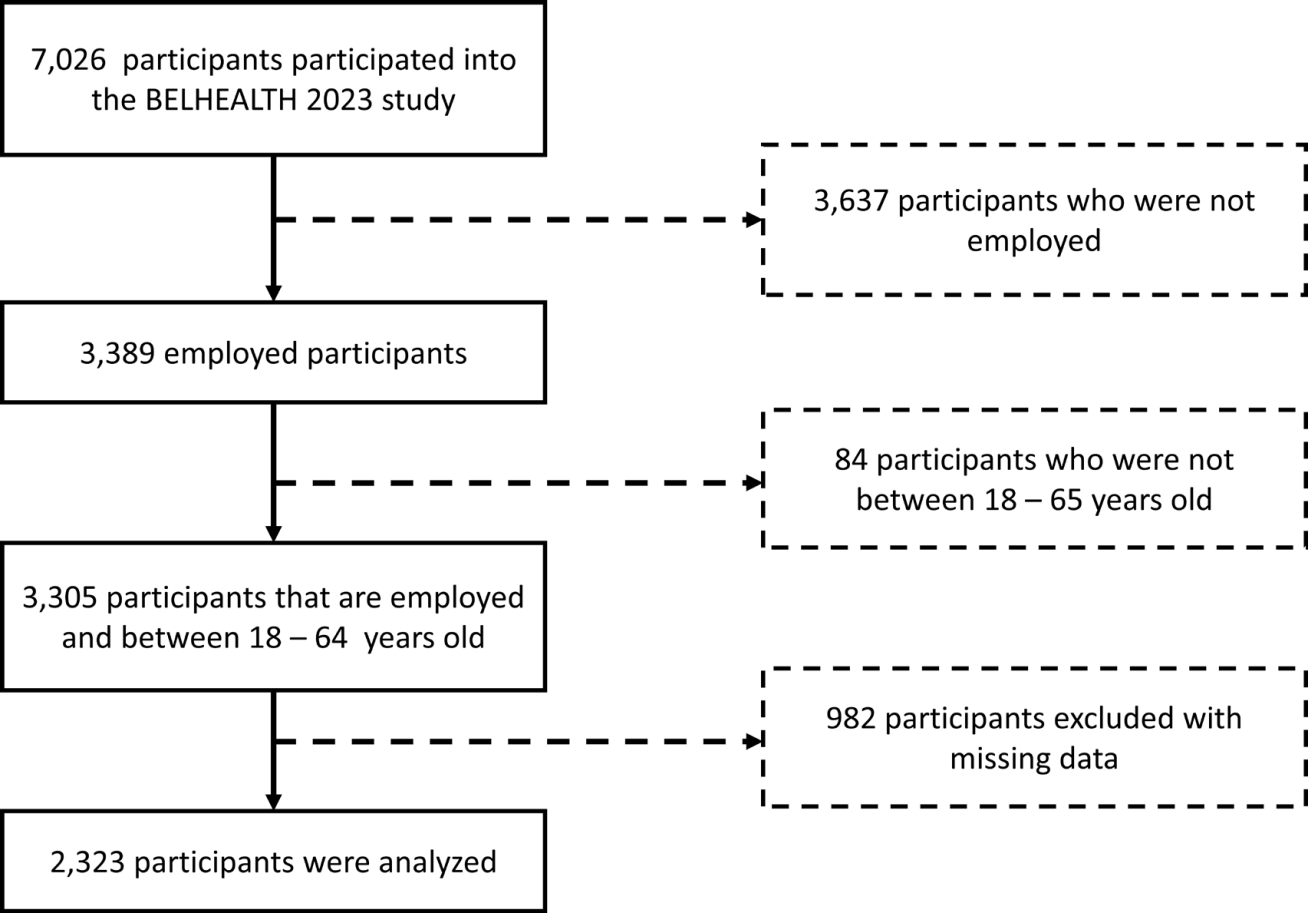
Participant inclusion and exclusion flow. In total, 7026 participants participated in the BELHEALTH June 2023 wave, leaving at the end 2323 participants (3797 participants were not employed, 84 were outside the age range 18–65 and 982 participants had missing data).

For this study, data from the survey wave of June 2023 were analysed. Participants were included if they were aged between 18 and 64 years and employed at the time of the questionnaire completion (figure 1). A complete case data analysis was performed and the key characteristics of the participants can be found in online supplemental material 2.

### Measurements

Telework served as the exposure variable, while workload, emotional load, role conflict, autonomy, social support and skills use were considered mediators. Burnout, work engagement, anxiety and depression were examined as outcome variables. Table 1 summarises the measurements used in this study.

**Table 1:** Variables included in the analysis of the relationship between telework and mental health

Variable name	Categories
Exposure	
Telework	Categorical variable based on the question: ‘Do you work from home?’ (0: non-teleworker, ‘Never, because this is not possible in my job’, or ‘Never, because I choose not to work from home’, 1: monthly teleworker (‘About one day a month’, ‘More than one day a month but less than one day a week’), 2: weekly teleworker (‘About one day a week’, ‘Several days a week’), 3: daily teleworker (‘daily*’*)).
Mediators	
Workload scale (Job demands)	Variable indicating whether the employee needs to perform certain tasks within a short time frame. Continuous average of three items (eg, ‘I have to hurry in my job’; Short Inventory to Monitor Psychosocial Hazards, SIMPH).^45^, sCA: 0.90.
Emotional load (Job demands)	Variable indicating whether the employee has emotional demanding tasks (eg, difficult conversations with clients). Continuous average of three items (eg, ‘I encounter touching situations through my work’) (SIMPH).^45^ sCA: 0.91
Role conflict (Job demands)	Variable indicating whether the employee encounters conflicting directions. Continuous average of three items (eg, ‘I get conflicting orders’) (SIMPH).^45^ sCA: 0.79.
Autonomy (Job resource)	Continuous average of three item autonomy scale a (eg, ‘I can influence my pace of work’) (SIMPH).^45^ sCA: 0.81.
Social support (Job resource)	Variable indicating the self-perceived support within the environment. Continuous average of six item social support scale (eg, ‘I can ask my colleagues for help if needed*’*) (SIMPH).^45^ sCA: 0.83.
Skills use (Job resource)	Variable indicating the self-perceived utilisation of one’s skills within the environment. Continuous average of a three item skills use (eg, ‘I learn new things at work’) (SIMPH).^45^ sCA: 0.85, three items.
Outcomes	
Anxiety	Binary variable measured using the Generalised Anxiety Disorder-7 (GAD-7) questionnaire 21, a widely used instrument that has been validated in English, Dutch and French. Responses can be coded as 0 for no anxiety, and 1 for generalised anxiety disorder (threshold of 10+ from the GAD-7).^46^ The indicator is calculated by summing the scores of each item on the scale, after recoding them to (0–3) where the value 0 represents ‘not at all’ and the value three represents ‘nearly every day’. The total score—ranging between 0 and 21—is then dichotomised, with the value 10+ being used to identify individuals with a likelihood of generalised anxiety disorder.^17^ sCA: 0.91, seven items.
Depression	Binary variable derived using the Patient Health Questionnaire-9, a widely used scale to screen for depression and assess the severity of depressive symptoms.^47^ Likelihood of depression is expressed with this binary variable (0: is no likelihood of, 1: likelihood of the depression).^17^ sCA: 0.88.
Burnout	Continuous variable assessed using the Burnout Assessment Tool-12 21, which measures burnout across four core dimensions: exhaustion, mental distance and emotional and cognitive impairment. An average burnout score was derived from those items, providing a continuous measure of burnout severity.^48^ sCA: 0.89.
Work engagement	Work engagement was measured using the Utrecht Work Engagement Scale-3 (UWES-3). The UWES-3 can be seen as a continuous variable, capturing : vigour, dedication and absorption. The average score was taken as a measure of work engagement calculated.^49^ sCA: 0.79.
Covariates	
Sex	Assigned at birth (1: male or 0: female)
Age	Continuous variable (expressed in years)
Education	The binary education variable distinguishes between individuals with a secondary degree (0) and those with a bachelor’s degree or above (1).
Type of contract	Binary variable (1: permanent contract, 0: temporary contract within the organisation, or temporary contract with an employment agency, or other)
Chronic illness	Self-reported presence of chronic illnesses or conditions (0 marked as lack of illness, 1 as present).
Functional limitation	Self-reported presence of chronic illnesses or conditions (0 marked as lack of illness, 1 as present).
Quality of social support	Continuous variable from poor, moderate, to strong social support.
Work skill	Job categories were asked to participants: (1) worker without qualification (eg, machine operator, production worker), (2) worker with qualification or team leader (eg, electrician, fitter, welder), (3) executive or administrative employee (eg, typist, secretary, shop clerk); (4) mid-level employee or supervisor (eg, computer programmer, teacher, sales representative), (5) higher-level employee or lower/middle-level manager (eg, office manager, engineer, lecturer), (6) senior executive or director (eg, head of department, senior manager, school head). For the analysis, these categories were collapsed into three levels: level 1 (categories 1 and 2), level 2 (categories 3 and 4) and level 3 (categories 5 and 6).

This table presents the variables considered in this study. Confounders are factors that may influence both the likelihood of engaging in telework and mental health status. Mediators represent potential pathways through which telework may impact mental health. Outcomes reflect the self-assessed psychological states. Covariates are individual and work-related characteristics adjusted for in the analysis. Calculated standardised Cronbach alpha (sCA).

### Statistical analysis

**Figure 2:**
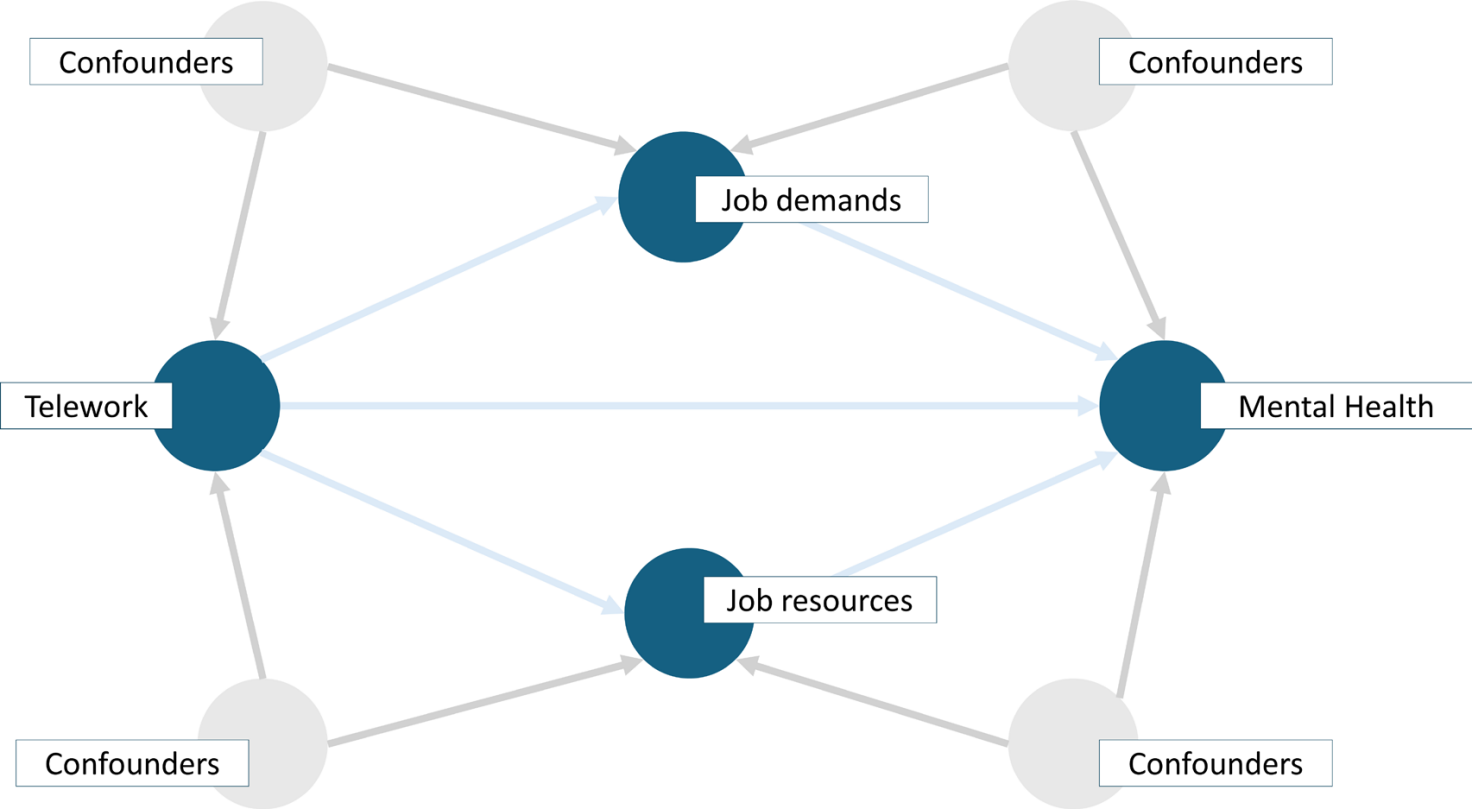
Direct acyclic diagram showing the hypothetical effect of telework on mental health. The graph represents telework (monthly, weekly, daily) as the exposure variable affecting mental health outcomes (anxiety, depression, burnout and work engagement) compared to non-telework. The direct effect is the direct link between telework on these outcomes, while lines through the job demands and resources indicate the individual indirect effects mediated through: autonomy, skills use, social support (job resources), workload, emotional load and role conflict (job demands). Additionally, confounders were: sex, age, education, type of contract, chronic illness, functional limitation, social support and work skill.

The statistical analyses are embedded in a causal inference framework, guided by a directed acyclic graph that formalises our assumptions about the data-generating process (figure 2). This approach was chosen because our primary aim was to estimate the impact of telework on mental health, while investigating potential mediators.^22 23^ Please note that causal interpretations from path analyses are limited, as they depend on assumptions that cannot be fully verified.

A mediation analysis estimates how an exposure variable *A* causally affects a dependent outcome variable *Y* through an intermediate variable, called a mediator *M*, that lies on the causal path from *A* to *Y*. The total effect of *A* on *Y* is decomposed into an indirect effect via *M* and a direct effect from *A* to *Y* that does not pass through *M*. In our study, we assumed that the exposure preceded the outcome and that the mediator occurs after the exposure but before the outcome.

Assuming an incorrect causal structure among the mediators can further bias the estimated effect of telework on mental health, therefore we used interventional effects models. These models can take into account multiple mediators and allow to derive causal (in)direct effect through each mediator without the need to specify any causal structure among the mediators.^24 25^ Hence, this method enables decomposing the effect of telework on mental health outcomes into a direct effect and indirect effects through each mediator separately. Given that telework is not randomised, we made the assumption that the relevant confounders were accounted for in the outcome and mediator models to preclude unmeasured confounding. When this unmeasured confounder assumption is violated, the derived effects become unidentifiable. Furthermore, causal indirect effects are often less robust than total and direct effects given that additional unconfoundness assumptions are added here. More details on interventional effects model and the underlying assumptions can be found in Loh *et al* (2021).^24 26^

Let us denote the exposure (telework) as *A*, with *z*=(0*,* 1*,* 2*,* 3) exposure levels (non-teleworkers, monthly, weekly or daily teleworkers) and let A_z_ (z=1, 2, 3) denote a dummy variable with *A*_z_=1 when *A*=z and 0 otherwise. We considered the following continuous mediators *M*: workload, emotional load, role conflict, autonomy, social support and skills use. Moreover, the outcome *Y* was either binary (anxiety, depression) or continuous (burnout, work engagement). Let *C* denote a vector of the confounder variables: sex, age, education, type of contract, chronic illness, functional limitation, chronic limitation, quality of social support and work skills. All of the variables except age and quality of social support were binary variables.

To estimate interventional direct and indirect effects, two sets of linear regression models were considered.

First, to estimate the total effect of each level z of the exposure on each outcome Y_q_ (*q=1, …, 4*), conditional on the confounders, we considered the following total effects model per outcome *Y* as a function of the confounders:


$$E\left(Y_{q}|C\right)=\delta_{0q}+\sum_{z=1}^{3}\delta_{1zq}A_{z}+\sum_{j=1}^{8}\delta_{2jq}C_{j}$$

Also, we modelled each outcome *Y* as a function of exposure A, and mediators; conditional on confounders *C*:


$$E\left(Y_{q}\;|\;A_{z},\;M_{s},\;C\right)=\;\beta_{0q}+\sum_{z=1}^{3}\beta_{1zq}A_{z}+\sum_{s=1}^{6}\beta_{2sq}M_{s}+\sum_{c=1}^{8}\beta_{3cq}C_{c}\;$$

Even with a binary outcome, we chose to employ linear models instead of generalised linear models, assuming a linear probability model. This strategy provided several benefits. First, it allowed for direct interpretation of the estimated effects in terms of probabilities. Moreover, linear models made our mediation analysis approach computationally feasible. In the online supplemental material 3, we added a sensitivity analysis with anxiety and depression expressed as total scores of the items.

Second, each mediator, *M_s_* (*s*=1*, …,* 6) was regressed on exposure *A*; conditional on confounders *C*:


$$E\left(M_{s}|A,C\right)=\theta_{0s}+\sum_{z=1}^{3}\theta_{1zs}A_{z}+\sum_{j=1}^{8}\theta_{2js}C_{j}$$

Here, we investigated the indirect effect (the effect through each mediator) and the joint indirect effect (the effect of all the mediators together).

Under the models above, the indirect effect of A on mental health outcome Y_q_, comparing A=z to A=0 (eg, comparing monthly telework to no telework), through mediator *M*_s_ was obtained by the product of coefficients method and equals $\theta_{1zs}\beta_{2zq}$.

The joint indirect effect was defined as the sum of the indirect effects across all mediators. This effect indicates a shift of the joint distribution of all the mediators under no telework to the joint distribution under the exposure (monthly, weekly, daily) changes either the likelihood (anxiety, depression), or the average score (burnout, work engagement).

Furthermore, the direct effect of A when comparing A=z to A=0 was defined as $\beta_{1zq}$. The total direct effect of A when comparing A=z to A=0 was captured by $\delta_{1zq}$ and it equals the sum of the indirect effects and direct effects.

The estimated (in)direct effects of the binary outcome models (anxiety, depression) are expressed in percentage (%) expressing a risk difference in outcome in the telework group (monthly, weekly or daily telework) compared with the non-telework group. For the continuous variable (burnout, work engagement), the effects are expressed in changes in the average outcome when changing non-telework to monthly, weekly or daily telework.

All effects were estimated using lavaan^27^ and can be interpreted as mediator-specific effects except for the total effects estimates.^25^

### Data management

R V.4.2.0 was used to perform the analyses.^28^ The analysis code and output of the code can be found on GitHub (https://github.com/Eduardo-a-bmo), along with the session info of the used packages. The data from this study is available on reasonable request.

## Results

### Study population

The data of a total of 2323 participants were analysed. The median age was 50.0 years (min: 23.0, max: 64.0). Table 2 summarises the demographic characteristics. Furthermore, the sample characteristics with the Belgian population characteristics of 2023 (based on STATBEL data) are compared in online supplemental material 4.

**Table 2:** Sample characteristics

	Non-teleworker (N=850)	Monthly teleworker (N=243)	Weekly teleworker (N=1157)	Daily teleworker (N=73)	Overall (N=2323)
Age					
Mean (SD)	48.6 (10.3)	48.1 (10.8)	48.4 (9.9)	51.9 (8.8)	48.5 (10.1)
Median (Min, Max)	50.0 (23.0, 64.0)	51.0 (25.0, 64.0)	50.0 (23.0, 64.0)	53.0 (30.0, 64.0)	50.0 (23.0, 64.0)
Sex					
Female	585 (68.8%)	151 (62.1%)	740 (64.0%)	40 (54.8%)	1516 (65.3%)
Education					
Bachelor or higher	641 (75.4%)	222 (91.4%)	1012 (87.5%)	66 (90.4%)	1941 (83.6%)
Work skill					
Lower/mid level	648 (76.2%)	113 (46.5%)	609 (52.6%)	39 (53.4%)	1409 (60.7%)
High level	202 (23.8%)	130 (53.5%)	548 (47.4%)	34 (46.6%)	914 (39.3%)
Type of contract					
Temporary or other	54 (6.4%)	20 (8.2%)	54 (4.7%)	3 (4.1%)	131 (5.6%)
Permanent	796 (93.6%)	223 (91.8%)	1103 (95.3%)	70 (95.9%)	2192 (94.4%)
Suffering from chronic illness					
Yes	562 (66.1%)	163 (67.1%)	779 (67.3%)	39 (53.4%)	1543 (66.4%)
Functional limitation					
Yes	665 (78.2%)	206 (84.8%)	932 (80.6%)	47 (64.4%)	1850 (79.6%)
Quality of social support					
Mean (SD)	2.09 (0.70)	2.23 (0.730)	2.11 (0.69)	1.86 (0.75)	2.10 (0.70)
Median (Min, Max)	2.00 (1.00, 3.00)	2.00 (1.00, 3.00)	2.00 (1.00, 3.00)	2.00 (1.00, 3.00)	2.00 (1.00, 3.00)
Anxiety					
High likelihood	115 (13.5%)	26 (10.7%)	142 (12.3%)	16 (21.9%)	299 (12.9%)
Depression					
High likelihood	85 (10.0%)	18 (7.4%)	110 (9.5%)	15 (20.5%)	228 (9.8%)
Burnout					
Mean (SD)	2.07 (0.61)	2.02 (0.55)	2.05 (0.634)	2.05 (0.68)	2.05 (0.62)
Median (Min, Max)	2.00 (1.00, 4.00)	2.00 (1.00, 3.58)	2.00 (1.00, 4.50)	1.92 (1.00, 3.75)	2.00 (1.00, 4.50)
Work engagement					
Mean (SD)	3.69 (0.75)	3.71 (0.64)	3.57 (0.79)	3.59 (0.75)	3.63 (0.76)
Median (Min, Max)	3.67 (1.00, 5.00)	3.67 (2.00, 5.00)	3.67 (1.00, 5.00)	3.67 (1.67, 5.00)	3.67 (1.00, 5.00)

Description of the sample characteristics across the four levels of telework (no teleworker, monthly telework, weekly telework, full-time telework) for the covariates, mediators and outcome values. Sex refers to assigned sex at birth (female or male). Work skill refers to a binary variable from three categories based on the job category (high-level, or mid-level, reference level). Education is a binary measure on high school or less completed education (reference level), and bachelor or higher. Type of contract is a binary measure between temporary contract or other (reference), or permanent contract. Suffering from chronic illness, and functional limitation indicate the presence of illness (reference level no illness). Quality of social support is expressed in poor, moderate and high support and expressed as a continuous variable. The continuous mediators were emotional support, workload, social support, role conflict, autonomy and skills use. The outcome variables were burnout, work engagement, depression and anxiety.

### Mediation analysis

**Figure 3:**
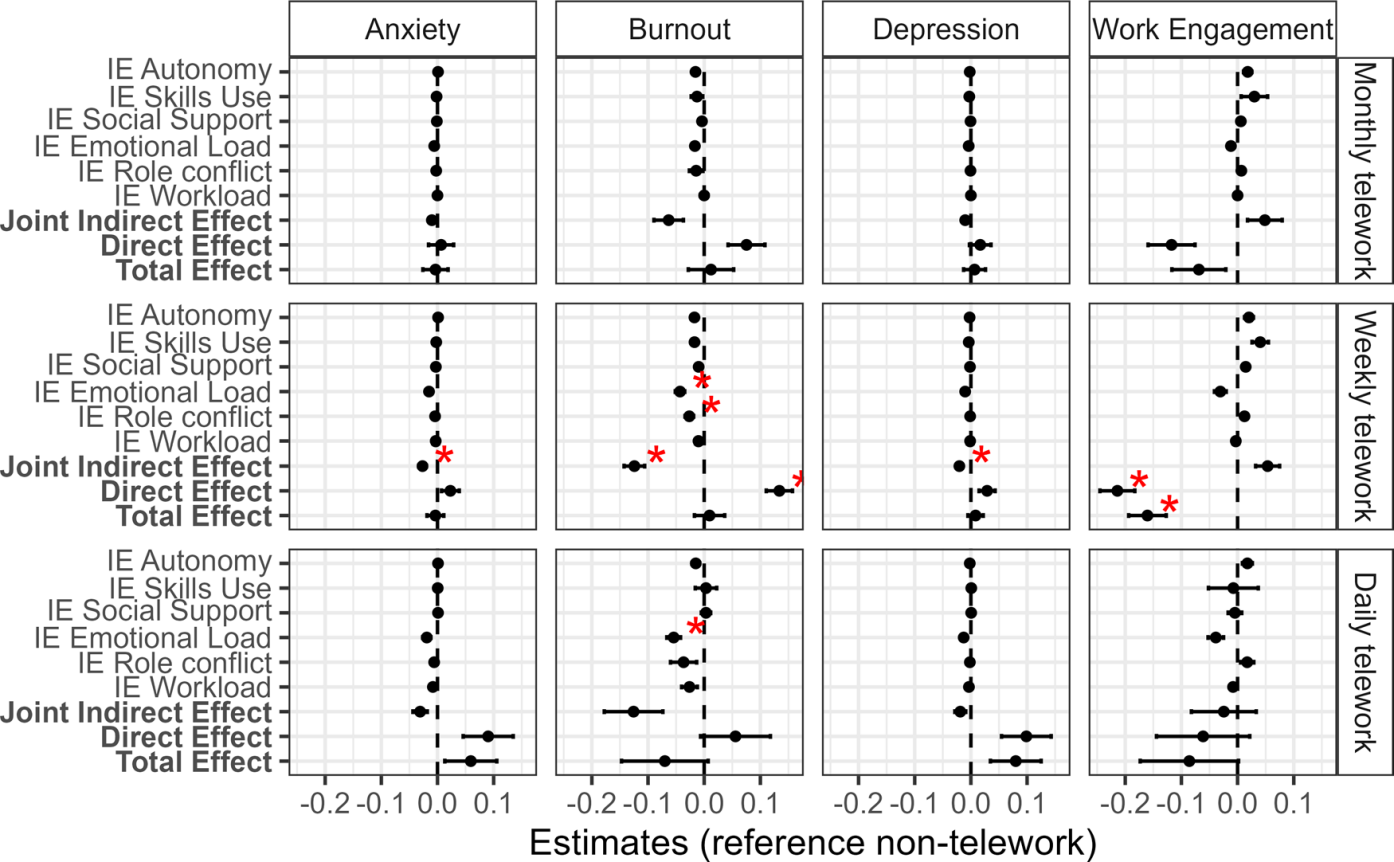
Interventional total, (in)direct and joint indirect effects across three levels of telework for the four mental health outcomes. The total, direct, indirect (IE) and joint indirect effects are shown per exposure of telework (monthly telework, N=243; weekly telework, N=1157; daily telework: N=73) and outcome (depression, anxiety, burnout and work engagement). The total effects are the sum of the indirect and direct effects. The direct effect is the estimate of the exposure on the outcome while controlling for confounders. Here, the different indirect effects are decomposed through the mediators: autonomy, skills use, social support, emotional load, role conflict and work load. The red asterisk indicates whether the value is significant after Bonferroni-Holm multiple comparison correction (alpha level=5%).

A mediation analysis with multiple mediators was conducted for each of the four outcomes (anxiety, depression, burnout, work engagement). The total effects, the interventional (in)direct effects and joint indirect effects are reported. For the exposure, non-telework served as the reference category against which the other telework categories were compared. Figure 3 summarises the results visually of the total, direct and (joint) indirect effects. SEs, p values and 95% CIs were constructed using 1000 non-parametric bootstrap samples. Adjustment for multiple comparisons was applied using the Bonferroni-Holm method per outcome with an alpha threshold of 0.05 (based on the p-adjusted values). In the online supplemental material 5, the estimated interventional effects for all the outcomes can be found.

#### The relationship of telework and anxiety

We did not observe any significant differences in anxiety across the teleworking groups (monthly, weekly, daily) compared with the non-teleworker group. More specifically, our results show that there is no significant total effect difference (−0.36%, 95% CI −4.80% to 4.43%; p >0.05) between monthly telework and no telework for anxiety. Similarly, this study could not identify a total effect difference between weekly telework and no telework on anxiety with an estimated difference of −0.38% (95% CI −3.38% to 2.79%; p >0.05). For daily telework, we found a non-significant total effect difference of 5.93% (95% CI −2.49% to 15.27%; p >0.05) in anxiety compared with the group that did not perform telework.

When looking at the disentangled effect of teleworking on anxiety into direct and indirect effects, we only observed a significant indirect effect through the combined effect of all mediators of −2.66% (95% CI −4.05% to −1.35%, p <0.05) when comparing weekly teleworkers to non-telework group. This difference implies a protective combined effect of the mediators with a 2.66% lower probability of anxiety in the weekly telework group as compared with the non-telework group. Regarding the other (joint) indirect effects, no statistically significant effects were found for monthly, weekly and daily telework exposure. Other results are presented in the online supplemental material 5.

#### The relationship of telework and depression

We did not find any significant differences in depression across the teleworking groups (monthly, weekly, daily) compared with the non-teleworker group. Here, the total effect of monthly telework was 0.0067 (95% CI −0.0329 to 0.0489, p>0.05), for weekly telework on depression it was 0.0083 (95% CI −0.0204 to 0.0341, p>0.05), or 8.30%) and for daily telework this effect was 0.0799 (95% CI −0.0044 to 0.1714; p>0.05), or 7.99%, is found.

When dividing the total effect into direct and indirect effect, we only found a significant joint indirect effect of weekly telework −0.0205 (95% CI −0.0314 to –0.0095; p<0.05). This indicates that when considering all the mediators together, there is a reduction of 2.05% depression by 2.05% compared with the non-telework group.

#### The relationship of telework and burnout

We did not find any significant differences in burnout across the teleworking groups (monthly, weekly, daily) compared with the non-teleworker group. In particular, the total effect of monthly telework on burnout was 0.0099 (95% CI −0.0698 to 0.0885, p>0.05), which indicated an increase in average burnout of 0.0099 in this group compared with the non-telework group. Similarly, the total effect of weekly telework on burnout was 0.0098 (95% CI −0.0433 to 0.0596, p>0.05). For the total effect of daily telework, a non-significant total effect equal to a reduction in the average burnout of −0.0701 (95% CI −0.2118 to 0.0769, p>0.05) is found.

When assessing the effect of teleworking on burnout separately through direct and indirect effects, we find significant indirect and direct effects in weekly telework compared with non-teleworkers. To be specific, the direct effects of weekly telework indicated that moving from no telework to weekly telework while keeping the mediator distributions fixed to those for the non-telework exposure, increased burnout significantly on average points by 0.1339 (95% CI 0.0875 to 0.1801; p<0.01). Daily telework increased burnout non-significantly by 0.0555 (95% CI −0.0597 to 0.1830; p>0.05).

Indirect effects showed reductions in the average burnout points in weekly telework mediated through: emotional load (−0.0427, 95% CI −0.0600 to –0.0272, p<0.01), role conflict (−0.0266, 95% CI −0.0419 to −0.0122, p<0.05). This indirect effect can be interpreted as the decreased effect of weekly telework (vs non-telework) on burnout through emotional load and role conflict. The indirect effect of emotional load in daily telework was also significant (−0.0540, 95% CI −0.0784 to −0.0330; p<0.01).

Furthermore, the joint indirect effects in weekly telework was −0.1241 (95% CI −0.1592 to –0.0879; p<0.01). This indirect effect indicates that shifting the joint distribution of the mediators under no telework to the joint distribution under weekly telework reduces burnout by 0.12 average points in the weekly telework group. In other words, we observed a reduction in burnout scores when considering all the mediators together.

#### The relationship of telework and work engagement

While evaluating the overall effect of telework on work engagement, we only found a significant decrease of weekly telework on work engagement (−0.1614, 95% CI −0.2286 to −0.0972; p<0.01). The total effect of monthly telework on work engagement indicated a non-significant increase in average work engagement of −0.0666 (95% CI −0.1548 to 0.0216, p>0.05). For the total effect of daily telework, a non-significant effect equal to a reduction in the average work engagement of −0.0861 (95% CI −0.2560 to 0.0712; p>0.05) is found.

When looking at the direct and indirect effect of telework, we did not find a significant effect of monthly telework on work engagement (−0.1159, 95% CI −0.1884 to −0.0354, p>0.05). In weekly telework, work engagement was reduced by −0.2158 (95% CI −0.2770 to –0.1561; p<0.01) on average. Daily telework did not have a significant impact on work engagement −0.0622 (95% CI −0.2248 to 0.002; p>0.05). The (joint) indirect effects on work engagement were mostly positive but statistically not significant.

## Discussion

In this study, we investigated whether the frequency of telework is (in)directly associated with mental health outcomes, including anxiety, depression, burnout and work engagement. We found a significant negative total effect of weekly telework on work engagement. Also, we found significant reductions of anxiety, depression and burnout when considering all the indirect effects together (joint indirect effects). Lastly, indirect effects of weekly telework through emotional load and role conflict seem to reduce burnout. See online supplemental table 6 for an overview of the key findings.

### Direct effects of telework on mental health

Telework was not significantly directly associated with an increase in anxiety nor depression, and was significantly directly associated with an increase in burnout, and a total effect reduction in work engagement. In contrast with our study, Afonso *et al.* (2021) found empirical evidence that telework might be directly associated with depression and anxiety.^29^ Their study also focused on full-time teleworkers at the start of telework during the COVID-19 pandemic, which might have aggravated the likelihood of depressive symptoms. Consistent with the findings of Sardeshmukh *et al*,^9^ a negative direct effect between telework and work engagement was observed in our study. However, in our study, this effect was only found in weekly telework. This may be linked to the limited impact of telework on participants who are teleworking at a lower frequency, as well as the small sample size observed in the daily telework group.

This direct link between telework and work engagement, and between telework and burnout can be attributed to the perception of telework as a job demand. Ample evidence demonstrates that job demands predict burnout and are negatively related to work engagement.^4 30^ In the context of telework, organisational and policy-related factors may transform telework into a demand (eg, voluntary or non-voluntary telework).

Kaduk *et al* (2019) studied the difference between voluntary and involuntary telework. The authors found that voluntary telework was associated with lower emotional exhaustion, whereas involuntary telework was associated with higher emotional exhaustion. These findings can be further explained by the self-determination theory (SDT)^31 32^ which states that the three main needs (competence, autonomy and relatedness) determine mental health. The SDT suggests that working in a controlled, externally regulated environment—where employees have little choice over telework arrangements—may harm well-being.^33^ Therefore, future research should evaluate whether the possibility to engage in telework (eg, voluntary, involuntary or not allowed) impacts mental health outcomes along with the extent of telework.

Furthermore, the direct relationship between telework and work engagement might be attributed to work centrality. Employees with high work centrality are characterised by high absorption and immersion in their work allocate resources to their work instead of allocating it to their private life. This is because the employee identifies more with the work role, and spends resources on work (eg, time). In the case of telework, employees who often are engaged with this practice might be thought to be less connected with their work roles, due to the lack of face-to-face interactions, etc. Therefore, this suggests that those individual evaluative factors further influence work engagement.^34^

### Indirect effects of telework on mental health

Telework seemed to have an indirect effect on mental health, mediated through various job demands and job resources. Here, indirect effects refer to the influence of telework via the mediators.^35^ Through the different job demands and job resources, telework seemed to indirectly affect anxiety, depression, burnout and work engagement.

The indirect effects of telework through emotional load may be explained by its potential to alleviate the stress and strain associated with emotional demands. Emotional demands, positively linked to higher risk of burnout and negatively to work engagement,^36 37^ may decrease in telework due to a lower frequency of intense personal interactions (eg, difficult client conversations). However, the absence of such challenges may reduce engagement by lowering the sense of accomplishment from difficult conversations.

The link of telework on mental health has been widely studied, but findings remain inconsistent. For instance, Vander Elst *et al* found no direct or indirect link between telework and work engagement.^16^ This discrepancy could be attributed to the operationalisation of telework, or the choice of the reference groups.

Many operationalisations of telework have been proposed (Framework Agreement of Telework, European Trade Union Confederation);^38^ however, there is no consensus on a standardised approach for measuring telework. In our study, the frequency of telework was expressed as the number of days per month and week, whereas Vander Elst *et al* expressed the extent of telework solely as the number of days per week.^16^ In contrast, Sardeshmukh *et al* expressed telework in terms of hours,^9^ whereas the study of Islam *et al* measured the shift from not teleworking to teleworking due to COVID-19 as a binary exposure.^39^ Allen *et al* argue that telework is not an ‘all-or-nothing’ practice, and thus the extent of telework is more relevant (eg, number of hours teleworked).^38^ Although the extent of telework matters, the number of hours worked per work location (eg, on the train, coworking space) might give a more accurate picture of the impact of telework. Research efforts are thus necessary to have a consensus-based measure of telework duration and location.

Similarly, the reference group is different across different studies. This study included non-teleworkers as a reference (individuals who chose not to telework or were unable to telework), while Sardeshmukh *et al* included those who telework 8 hours a week as a reference group.^9^ Vander Elst *et al* included employees who never teleworked as a reference group.^16^ Such differences in reference groups may contribute to the variability in findings across the literature. To improve comparability, markers such as the teleworkability index^40^ could be used as an alternative definition of telework to compare mental health across different job types.

### Strengths and limitations

There are two main strengths in this study. The first strength is that we did not specify an underlying causal structure among the mediators, an approach that is particularly advantageous when there is a lack of evidence in the causal relationships between mediators. Contrarily to natural effect models, a different definition of indirect and direct effects is derived where the underlying causal structure is left unspecified.^24 41 42^ Although the interventional effects model has its advantage that the relationship between mediators is left unspecified, it does not provide further information on the structure between the mediators. Further research could be expanded to allow for interactions between telework, and the mediators, and the mediators themselves. The second strength is the large sample size (n=2327) which comes with a heterogeneous sample.

This study has several limitations. First, it relies on a cross-sectional dataset, with the primary assumption being that participants had been teleworking for some time before completing the survey (during COVID-19). In a cross-sectional study, temporality can be assessed with difficulty, unless the exposure variable clearly precedes the outcome (eg, a genetic factor). Second, the sample is not fully representative of the Belgian population and the results might only be partially generalisable.^43^ Third, interventional analyses assume the absence of unmeasured confounders. Moreover, the causal relationship between telework and mental health remains uncertain due to inconsistent findings across studies. Future studies should systematically focus on effect modification across well-defined subgroups in sufficiently powered samples (eg, household composition^44^). Fourth, the subgroup of daily teleworkers in the sample is relatively small (n=73), resulting in wide confidence intervals and low power in those inferences.

Fifth, reverse causation cannot be ruled out. Employees’ mental and physical health may influence the decision to uptake telework, rather than the other way around. For instance, workers with burnout, depression or anxiety symptoms might stay at home to reduce exhaustion from commute or social interactions, and socially anxious employees might prefer to avoid face-to-face interactions. Future research should therefore examine the determinants of telework uptake and how this process influences the associations between telework and mental health. Finally, most of the effects that were found were of small magnitude, suggesting that other job characteristics have a greater influence on mental health than telework.

## Conclusions

This study contributes to the current literature on the relationship between telework and mental health. Job characteristics (eg, autonomy, skills use) are essential to understand how a work environment can affect a teleworker’s health. Although telework seems to be directly linked to an increase in burnout, and a decrease in work engagement, it also seems to have an indirect positive effect on those outcomes. When considering all the effects together (total effect), telework seems to impact work engagement negatively. Therefore, telework may have both a negative and positive effect on mental health. To cultivate a healthy workplace, it is essential to rethink these psychological aspects.

## Supplementary material

10.1136/bmjph-2025-003249 online supplemental file 1

10.1136/bmjph-2025-003249 online supplemental file 2

10.1136/bmjph-2025-003249 online supplemental file 3

10.1136/bmjph-2025-003249 online supplemental file 4

10.1136/bmjph-2025-003249 online supplemental file 5

10.1136/bmjph-2025-003249 online supplemental file 6

## Data Availability

Data are available upon reasonable request.
