# Supplementary material for: Exploring the mediating factors in the telework-mental health relationship: a cross-sectional analysis of the BELHEALTH study
Source: BMJ Public Health. 2026 Feb 18;4(1):e003249. doi: 10.1136/bmjph-2025-003249 (PMC12927397; doi:10.1136/bmjph-2025-003249)
Supplement: online supplemental file 2 [file bmjph-4-1-s002.docx]

# Table S2. Missing data per variable

| variable | n | Number.of.missings | Number.of.observations | Missing.percentage |
| --- | --- | --- | --- | --- |
| Anxiety | 3,305 | 15 | 3,290 | 0.5 |
| Depression | 3,305 | 16 | 3,289 | 0.5 |
| Burnout | 3,305 | 294 | 3,011 | 8.9 |
| Work Engagement | 3,305 | 163 | 3,142 | 4.9 |
| Sex | 3,305 | 0 | 3,305 | 0.0 |
| Age | 3,305 | 0 | 3,305 | 0.0 |
| Education | 3,305 | 1 | 3,304 | 0.0 |
| Regio | 3,305 | 34 | 3,271 | 1.0 |
| Work Skill | 3,305 | 129 | 3,176 | 3.9 |
| Type of Contract | 3,305 | 352 | 2,953 | 10.7 |
| Suffering from Chronic illness | 3,305 | 91 | 3,214 | 2.8 |
| Functional limitation | 3,305 | 67 | 3,238 | 2.0 |
| Quality of Social Support | 3,305 | 79 | 3,226 | 2.4 |
| Telework Categories | 3,305 | 87 | 3,218 | 2.6 |
| Emotional Load | 3,305 | 143 | 3,162 | 4.3 |
| Time Pressure | 3,305 | 122 | 3,183 | 3.7 |
| Role Conflict | 3,305 | 236 | 3,069 | 7.1 |
| Social Support | 3,305 | 270 | 3,035 | 8.2 |
| Skills Use | 3,305 | 138 | 3,167 | 4.2 |
| Autonomy | 3,305 | 111 | 3,194 | 3.4 |

# Figure S1. Missing data pattern


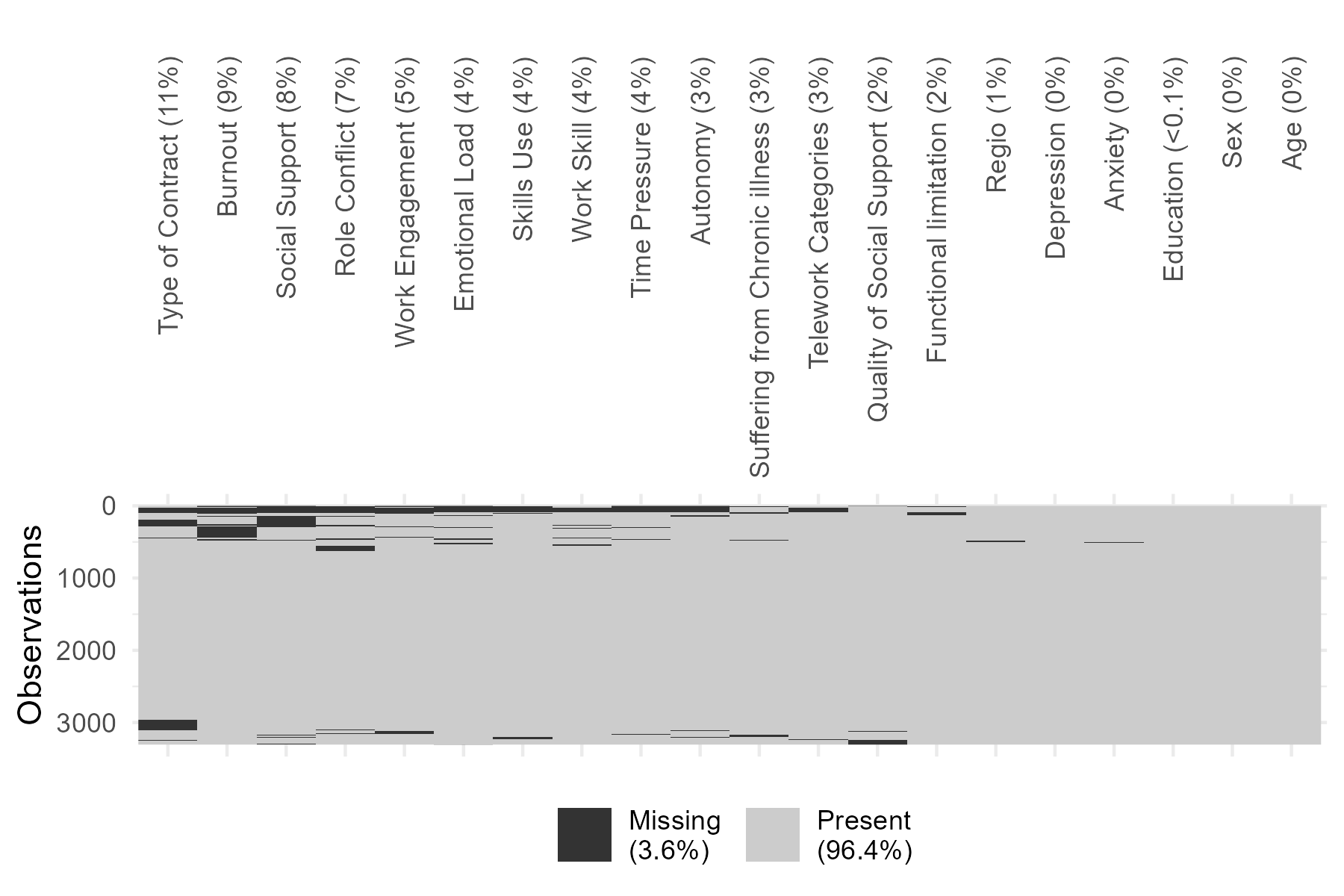


# Figure S2. Missing data pattern per telework category


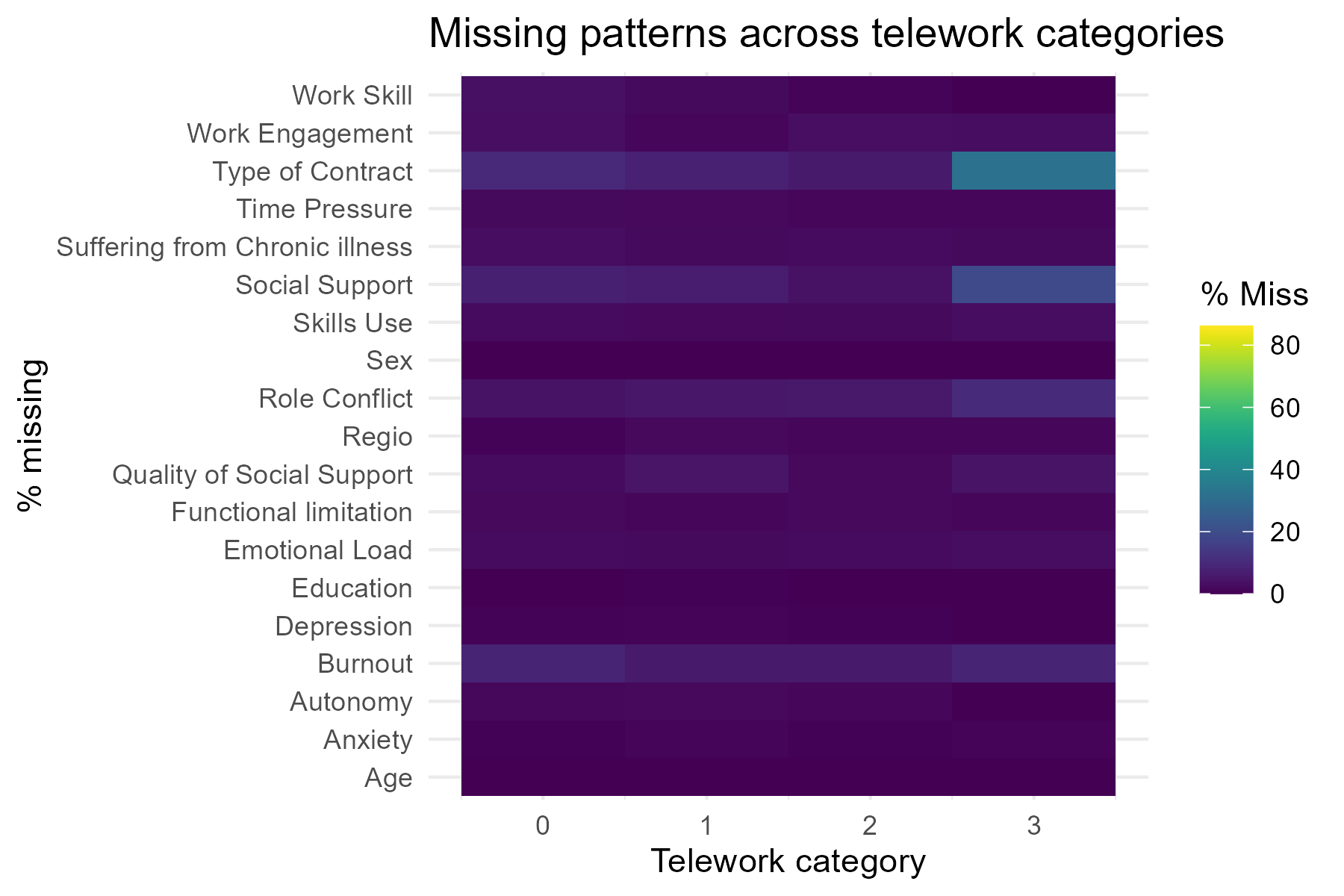


# Table. Missing data per outcome: Anxiety

|  | Observed | Missing | Overall |
| --- | --- | --- | --- |
|  | (N=3290) | (N=15) | (N=3305) |
| Sex |  |  |  |
| Male | 1115 (33.9%) | 5 (33.3%) | 1120 (33.9%) |
| Female | 2175 (66.1%) | 10 (66.7%) | 2185 (66.1%) |
| Age |  |  |  |
| Mean (SD) | 48.8 (10.3) | 52.7 (11.8) | 48.8 (10.3) |
| Median [Min, Max] | 51.0 [23.0, 64.0] | 57.0 [24.0, 63.0] | 51.0 [23.0, 64.0] |
| Education |  |  |  |
| High school  or lower | 553 (16.8%) | 4 (26.7%) | 557 (16.9%) |
| Bachelor or higher | 2736 (83.2%) | 11 (73.3%) | 2747 (83.1%) |
| Missing | 1 (0.0%) | 0 (0%) | 1 (0.0%) |
| Work Skill |  |  |  |
| Lower/Mid level | 1838 (55.9%) | 13 (86.7%) | 1851 (56.0%) |
| High level | 1325 (40.3%) | 0 (0%) | 1325 (40.1%) |
| Missing | 127 (3.9%) | 2 (13.3%) | 129 (3.9%) |
| Type of Contract |  |  |  |
| Permanent | 2762 (84.0%) | 14 (93.3%) | 2776 (84.0%) |
| Temporary  or other | 177 (5.4%) | 0 (0%) | 177 (5.4%) |
| Missing | 351 (10.7%) | 1 (6.7%) | 352 (10.7%) |
| Suffering from Chronic illness |  |  |  |
| Yes | 2120 (64.4%) | 6 (40.0%) | 2126 (64.3%) |
| No | 1080 (32.8%) | 8 (53.3%) | 1088 (32.9%) |
| Missing | 90 (2.7%) | 1 (6.7%) | 91 (2.8%) |
| Functional limitation |  |  |  |
| No | 670 (20.4%) | 4 (26.7%) | 674 (20.4%) |
| Yes | 2553 (77.6%) | 11 (73.3%) | 2564 (77.6%) |
| Missing | 67 (2.0%) | 0 (0%) | 67 (2.0%) |
| Quality of Social Support |  |  |  |
| Mean (SD) | 2.09 (0.703) | 1.93 (0.730) | 2.09 (0.703) |
| Median [Min, Max] | 2.00 [1.00, 3.00] | 2.00 [1.00, 3.00] | 2.00 [1.00, 3.00] |
| Missing | 78 (2.4%) | 1 (6.7%) | 79 (2.4%) |

# Table. Missing data per outcome: Depression

|  | Observed | Missing | Overall |
| --- | --- | --- | --- |
|  | (N=3289) | (N=16) | (N=3305) |
| Sex |  |  |  |
| Male | 1117 (34.0%) | 3 (18.8%) | 1120 (33.9%) |
| Female | 2172 (66.0%) | 13 (81.3%) | 2185 (66.1%) |
| Age |  |  |  |
| Mean (SD) | 48.8 (10.3) | 51.6 (8.14) | 48.8 (10.3) |
| Median [Min, Max] | 51.0 [23.0, 64.0] | 54.0 [29.0, 62.0] | 51.0 [23.0, 64.0] |
| Education |  |  |  |
| High school  or lower | 552 (16.8%) | 5 (31.3%) | 557 (16.9%) |
| Bachelor or higher | 2736 (83.2%) | 11 (68.8%) | 2747 (83.1%) |
| Missing | 1 (0.0%) | 0 (0%) | 1 (0.0%) |
| Work Skill |  |  |  |
| Lower/Mid level | 1841 (56.0%) | 10 (62.5%) | 1851 (56.0%) |
| High level | 1321 (40.2%) | 4 (25.0%) | 1325 (40.1%) |
| Missing | 127 (3.9%) | 2 (12.5%) | 129 (3.9%) |
| Type of Contract |  |  |  |
| Permanent | 2763 (84.0%) | 13 (81.3%) | 2776 (84.0%) |
| Temporary  or other | 177 (5.4%) | 0 (0%) | 177 (5.4%) |
| Missing | 349 (10.6%) | 3 (18.8%) | 352 (10.7%) |
| Suffering from Chronic illness |  |  |  |
| Yes | 2118 (64.4%) | 8 (50.0%) | 2126 (64.3%) |
| No | 1083 (32.9%) | 5 (31.3%) | 1088 (32.9%) |
| Missing | 88 (2.7%) | 3 (18.8%) | 91 (2.8%) |
| Functional limitation |  |  |  |
| No | 669 (20.3%) | 5 (31.3%) | 674 (20.4%) |
| Yes | 2553 (77.6%) | 11 (68.8%) | 2564 (77.6%) |
| Missing | 67 (2.0%) | 0 (0%) | 67 (2.0%) |
| Quality of Social Support |  |  |  |
| Mean (SD) | 2.09 (0.702) | 1.67 (0.816) | 2.09 (0.703) |
| Median [Min, Max] | 2.00 [1.00, 3.00] | 1.00 [1.00, 3.00] | 2.00 [1.00, 3.00] |
| Missing | 78 (2.4%) | 1 (6.3%) | 79 (2.4%) |

# Table. Missing data per outcome: Burnout

|  | Observed | Missing | Overall |
| --- | --- | --- | --- |
|  | (N=3011) | (N=294) | (N=3305) |
| Sex |  |  |  |
| Male | 1033 (34.3%) | 87 (29.6%) | 1120 (33.9%) |
| Female | 1978 (65.7%) | 207 (70.4%) | 2185 (66.1%) |
| Age |  |  |  |
| Mean (SD) | 48.8 (10.2) | 49.1 (11.0) | 48.8 (10.3) |
| Median [Min, Max] | 51.0 [23.0, 64.0] | 52.0 [23.0, 64.0] | 51.0 [23.0, 64.0] |
| Education |  |  |  |
| High school  or lower | 489 (16.2%) | 68 (23.1%) | 557 (16.9%) |
| Bachelor or higher | 2521 (83.7%) | 226 (76.9%) | 2747 (83.1%) |
| Missing | 1 (0.0%) | 0 (0%) | 1 (0.0%) |
| Work Skill |  |  |  |
| Lower/Mid level | 1714 (56.9%) | 137 (46.6%) | 1851 (56.0%) |
| High level | 1250 (41.5%) | 75 (25.5%) | 1325 (40.1%) |
| Missing | 47 (1.6%) | 82 (27.9%) | 129 (3.9%) |
| Type of Contract |  |  |  |
| Permanent | 2584 (85.8%) | 192 (65.3%) | 2776 (84.0%) |
| Temporary  or other | 164 (5.4%) | 13 (4.4%) | 177 (5.4%) |
| Missing | 263 (8.7%) | 89 (30.3%) | 352 (10.7%) |
| Suffering from Chronic illness |  |  |  |
| Yes | 1975 (65.6%) | 151 (51.4%) | 2126 (64.3%) |
| No | 990 (32.9%) | 98 (33.3%) | 1088 (32.9%) |
| Missing | 46 (1.5%) | 45 (15.3%) | 91 (2.8%) |
| Functional limitation |  |  |  |
| No | 612 (20.3%) | 62 (21.1%) | 674 (20.4%) |
| Yes | 2369 (78.7%) | 195 (66.3%) | 2564 (77.6%) |
| Missing | 30 (1.0%) | 37 (12.6%) | 67 (2.0%) |
| Quality of Social Support |  |  |  |
| Mean (SD) | 2.10 (0.700) | 1.94 (0.712) | 2.09 (0.703) |
| Median [Min, Max] | 2.00 [1.00, 3.00] | 2.00 [1.00, 3.00] | 2.00 [1.00, 3.00] |
| Missing | 72 (2.4%) | 7 (2.4%) | 79 (2.4%) |

# Table. Missing data per outcome: Work Engagement

|  | Observed | Missing | Overall |
| --- | --- | --- | --- |
|  | (N=3142) | (N=163) | (N=3305) |
| Sex |  |  |  |
| Male | 1066 (33.9%) | 54 (33.1%) | 1120 (33.9%) |
| Female | 2076 (66.1%) | 109 (66.9%) | 2185 (66.1%) |
| Age |  |  |  |
| Mean (SD) | 48.7 (10.2) | 49.8 (11.3) | 48.8 (10.3) |
| Median [Min, Max] | 51.0 [23.0, 64.0] | 53.0 [24.0, 64.0] | 51.0 [23.0, 64.0] |
| Education |  |  |  |
| High school  or lower | 521 (16.6%) | 36 (22.1%) | 557 (16.9%) |
| Bachelor or higher | 2620 (83.4%) | 127 (77.9%) | 2747 (83.1%) |
| Missing | 1 (0.0%) | 0 (0%) | 1 (0.0%) |
| Work Skill |  |  |  |
| Lower/Mid level | 1794 (57.1%) | 57 (35.0%) | 1851 (56.0%) |
| High level | 1290 (41.1%) | 35 (21.5%) | 1325 (40.1%) |
| Missing | 58 (1.8%) | 71 (43.6%) | 129 (3.9%) |
| Type of Contract |  |  |  |
| Permanent | 2695 (85.8%) | 81 (49.7%) | 2776 (84.0%) |
| Temporary  or other | 172 (5.5%) | 5 (3.1%) | 177 (5.4%) |
| Missing | 275 (8.8%) | 77 (47.2%) | 352 (10.7%) |
| Suffering from Chronic illness |  |  |  |
| Yes | 2052 (65.3%) | 74 (45.4%) | 2126 (64.3%) |
| No | 1042 (33.2%) | 46 (28.2%) | 1088 (32.9%) |
| Missing | 48 (1.5%) | 43 (26.4%) | 91 (2.8%) |
| Functional limitation |  |  |  |
| No | 642 (20.4%) | 32 (19.6%) | 674 (20.4%) |
| Yes | 2470 (78.6%) | 94 (57.7%) | 2564 (77.6%) |
| Missing | 30 (1.0%) | 37 (22.7%) | 67 (2.0%) |
| Quality of Social Support |  |  |  |
| Mean (SD) | 2.09 (0.702) | 2.08 (0.719) | 2.09 (0.703) |
| Median [Min, Max] | 2.00 [1.00, 3.00] | 2.00 [1.00, 3.00] | 2.00 [1.00, 3.00] |
| Missing | 70 (2.2%) | 9 (5.5%) | 79 (2.4%) |
