# Supplementary material for: Exploring the mediating factors in the telework-mental health relationship: a cross-sectional analysis of the BELHEALTH study
Source: BMJ Public Health. 2026 Feb 18;4(1):e003249. doi: 10.1136/bmjph-2025-003249 (PMC12927397; doi:10.1136/bmjph-2025-003249)
Supplement: online supplemental file 3 [file bmjph-4-1-s003.docx]

# Anxiety Total

# Depression Total
