# Supplementary material for: Exploring the mediating factors in the telework-mental health relationship: a cross-sectional analysis of the BELHEALTH study
Source: BMJ Public Health. 2026 Feb 18;4(1):e003249. doi: 10.1136/bmjph-2025-003249 (PMC12927397; doi:10.1136/bmjph-2025-003249)
Supplement: online supplemental file 4 [file bmjph-4-1-s004.docx]

**Table S1. Representativity comparison between the STATBEL data on telework and the BELHEALTH 2024 sample**

|  | **BELHEALTH 2023** | | **STATBEL 2023** |
| --- | --- | --- | --- |
|  | **Non-teleworker** | **Teleworker** | **Teleworker** |
| **Sex** |  |  |  |
| Men | 265 (31.2%) | 542 (36.8%) | 327,659 (29.9%) |
| Female | 585 (68.8%) | 931 (63.2%) | 325,145 (34.7%) |
| **Education Level** |  |  |  |
| Low/Middle education | 209 (24.6%) | 173 (11.7%) | 109,001 (14.7%) |
| High education | 641 (75.4%) | 1,300 (88.3%) | 543,804 (53.0%) |
