# Supplementary material for: Exploring the mediating factors in the telework-mental health relationship: a cross-sectional analysis of the BELHEALTH study
Source: BMJ Public Health. 2026 Feb 18;4(1):e003249. doi: 10.1136/bmjph-2025-003249 (PMC12927397; doi:10.1136/bmjph-2025-003249)
Supplement: online supplemental file 6 [file bmjph-4-1-s006.docx]

|  | **Anxiety** | **Depression** | **Burnout** | **Work engagement** |
| --- | --- | --- | --- | --- |
| **Total effect** | No effect | No effect | No effect | Reduction of work engagement  (weekly telework) |
|  |  |  |  |  |
| **Direct Effect** | No effect | No effect | Increase in burnout  (weekly telework) | Reduction of work engagement  (weekly telework) |
|  |  |  |  |  |
| **Indirect effect** | No effect | No effect | Reduction in burnout through role conflict  (weekly telework), and emotional load (weekly and daily telework) | No effect |
| **Joint Indirect effect** | Small effect (reduction of anxiety, weekly telework) | Small effect (reduction of depression, weekly telework) | Reduction in burnout  (weekly telework) | No effect |

**Table 3. Summary of the main findings.**
